# Real-World Evaluation of Early Initiation of Dapagliflozin and Sitagliptin Combination Therapy in the Management of Type 2 Diabetes Mellitus (REALIZE Study)

**DOI:** 10.7759/cureus.93632

**Published:** 2025-10-01

**Authors:** Sambit Das, A Ramachandran, SK Wangnoo, Prasun Deb, Yogesh Kadam, Abhijit Pednekar, Rohan N Kesarkar

**Affiliations:** 1 Endocrinology, Diabetes and Metabolism, Endeavour Clinics, Bhubaneswar, IND; 2 Diabetes and Endocrinology, Dr. A. Ramachandran's Diabetes Hospitals, Chennai, IND; 3 Diabetes and Endocrinology, Indraprastha Apollo Hospitals, New Delhi, IND; 4 Endocrinology, Krishna Institute of Medical Sciences, Secunderabad, IND; 5 Endocrinology, Poona Diabetes Centre, Pune, IND; 6 Scientific Services, USV Pvt Ltd., Mumbai, IND; 7 Diabetes and Endocrinology, Scientific Services, USV Pvt Ltd., Mumbai, IND

**Keywords:** central adiposity, comorbid conditions, dapagliflozin, early initiation, fixed dose combination, glycemic parameters, renal function, sitagliptin, type 2 diabetes mellitus (dm)

## Abstract

Background

Fixed-dose combinations (FDCs) of antidiabetic agents offer improved compliance and metabolic control in real-world settings.

Objectives

The present study assessed the effectiveness and safety of early initiation of an FDC of dapagliflozin and sitagliptin in Indian patients with type 2 diabetes mellitus (T2DM) and associated cardiometabolic comorbidities.

Methodology

This retrospective, observational study included patients aged 18-59 years with T2DM, baseline BMI ≥25 kg/m², HbA1c levels between 7.0% and 10.5%, and prescribed the FDC of dapagliflozin and sitagliptin across five Indian centers. Effectiveness outcomes included changes in glycemic profile, renal function, body weight, BMI, serum triglycerides (TGs), and blood pressure (BP).

Results

A total of 250 patients were included, with a mean age of 46.9 years, predominantly male. The mean waist circumference of 94.6 cm indicated central adiposity. Dyslipidemia and hypertension were the most common comorbidities. A significant reduction in glycated hemoglobin (HbA1c) was observed (-1.1%, p<0.001). Improvements in fasting plasma glucose (FPG) and postprandial plasma glucose (PPG) were statistically significant. Patients with dyslipidemia or hypertension also experienced significant reductions. The study further demonstrated significant improvements in renal parameters. Body weight and BP were significantly reduced with this FDC. No adverse events were reported.

Conclusion

Early initiation of an FDC of dapagliflozin and sitagliptin led to significant improvements in glycemic, renal, and cardiovascular parameters in patients with T2DM. Its favorable effectiveness and safety profile, even among those with common comorbidities such as hypertension and dyslipidemia, underscore its potential as a comprehensive treatment option in routine clinical practice.

## Introduction

Type 2 diabetes mellitus (T2DM) is a chronic metabolic disorder that significantly contributes to global mortality and morbidity, with an alarming rise in prevalence in low- and middle-income economies, especially India [1]. Abdominal obesity, commonly observed in South Asian populations, is strongly associated with systemic inflammation, insulin resistance, and lipid abnormalities [2]. Its increasing prevalence contributes to the growing burden of metabolic diseases. A study by Gupta RD et al. reported a positive correlation between abdominal obesity and the risk of T2DM in the Indian population [3]. In real-world settings, Indian patients with T2DM generally present with co-existing conditions such as hypertension and dyslipidemia [4]. The presence of these cardiometabolic conditions is significantly associated with an increased risk of cardiorenal complications. These co-existing conditions necessitate a treatment approach that goes beyond glycemic control and addresses overall cardiometabolic health. Hence, careful selection of glucose-lowering agents is vital for reducing long-term diabetes-related complications [5].

Although traditional recommendations advocate stepwise additions to metformin for achieving target glycated hemoglobin (HbA1c), the 2024 American Diabetes Association guidelines emphasize timely treatment intensification or modification in patients not meeting glycemic targets. These guidelines recommend a patient-centric approach that incorporates clinical factors including obesity, established atherosclerotic cardiovascular disease (ASCVD) or indicators of high ASCVD risk, alongside safety, tolerability, accessibility, usability, and cost in the selection of glucose-lowering agents [6]. Despite initial clinical inertia in adopting newer antidiabetic agents such as SGLT-2 and DPP-4 inhibitors, growing awareness of their glycemic, cardiovascular, and renal benefits has led to a paradigm shift in current treatment practices. A global survey including physicians from India, evaluating the causes of clinical inertia in T2DM cardiorenal risk management, reported that more than 80% of respondents supported early intensification. This notable shift in practice underscores the recognition of the comprehensive cardiometabolic benefits associated with newer antidiabetic agents [7].

Additionally, recent evidence suggests that South Asians, including Indians, may respond differently to standard antidiabetic agents. Sivadas A et al. reported that Indians and South Asians have a significantly higher prevalence of alleles responsible for reduced response to metformin. This could potentially contribute to suboptimal glycemic control with conventional first-line antidiabetic therapies, underscoring the need for additional treatment strategies [8].

Among the newer classes of glucose-lowering therapeutic agents, sodium-glucose cotransporter-2 (SGLT-2) inhibitors and dipeptidyl peptidase-4 (DPP-4) inhibitors have gained prominence in clinical practice due to their glycemic and pleiotropic effects [9]. Dapagliflozin, an SGLT-2 inhibitor, promotes glucose-lowering by blocking the reabsorption of filtered glucose in the kidneys and promoting urinary glucose excretion. In addition to glycemic effects, it offers extra-glycemic benefits such as weight loss, modest reductions in blood pressure (BP), and cardiovascular and renal protection [2]. Sitagliptin, a DPP-4 inhibitor, is known for improving glycemic profile, BP, lipid levels, and quality of life (QoL), with a lower incidence of hypoglycemia [10]. Furthermore, studies have indicated that the glucose-lowering efficacy of SGLT-2 and DPP-4 inhibitors is higher in Asians compared with Western populations [11].

The combination therapy of SGLT-2 and DPP-4 inhibitors has been reported to be both effective and safe [12]. In real-world settings, polypharmacy is commonly encountered in patients with T2DM, making fixed-dose combinations (FDCs) a rational strategy to improve adherence and reduce pill burden. By integrating complementary mechanisms of action, FDCs provide synergistic benefits along with reduced pill burden, improved tolerability, and cost-effectiveness [13]. The FDC of dapagliflozin and sitagliptin has been reported to be effective in reducing blood glucose levels and BMI in the Indian population with T2DM [14]. While certain trials and studies have demonstrated the efficacy and safety of this combination, data on its real-world effectiveness in patients with comorbid conditions in Indian clinical practice remain limited.

The present study aims to assess the impact of early initiation of the FDC of dapagliflozin and sitagliptin in Indian patients with T2DM and coexisting cardiometabolic conditions. The findings are expected to provide insights into the clinical effectiveness of this combination and support early treatment intensification in this high-risk population.

## Materials and methods

Study design and ethics

This retrospective, real-world study analyzed anonymized medical records from five (05) sites in metro cities (Hyderabad, Orissa, Pune, Delhi, and Chennai) across India. Site selection was guided by the footfall of diabetes patients and the accessibility of diagnostic testing. The primary aim was to evaluate the effect of early initiation of an FDC of dapagliflozin and sitagliptin in the management of T2DM. It included patients who met the eligibility criteria and were prescribed this FDC. Data retrieval was carried out using dedicated case report forms (CRFs). Ethics approval for conducting the study was obtained from the Shah Lifeline Hospital and Heart Institute Ethics Committee on June 8, 2024 (ECR/1588/Inst/MH/2021). Site-level approvals were also sought from the participating medical practitioners.

Study population

The study included male and female patients aged 18 to 59 years with T2DM, a baseline BMI ≥25 kg/m², HbA1c levels between 7.0% and 10.5%, and who were prescribed the dapagliflozin and sitagliptin FDC. Patients were excluded if their medical records were incomplete (e.g., missing baseline characteristics or treatment details) or, at the investigator’s discretion, if they had any serious underlying condition such as malignancy that could confound the study outcomes. The prescription visit of dapagliflozin and sitagliptin FDC was considered the baseline (visit 1), and the visit within 112 ± 20 days of baseline was considered the follow-up or end-of-study visit.

Study outcomes

The primary endpoints were the mean changes in glycemic parameters (HbA1c, fasting plasma glucose (FPG), and postprandial plasma glucose (PPG)). The secondary endpoints were the mean changes in body weight, BMI, blood pressure (BP), estimated glomerular filtration rate (eGFR), serum creatinine, urine albumin-creatinine ratio (uACR), and serum triglyceride (TGs) levels. The study also assessed the incidence of adverse events at the end of the study visit.

Statistical analysis

Statistical analysis was carried out using the SPSS, version 23. Missing data were not imputed. Baseline characteristics such as age, weight, height, BMI, waist circumference, and others were summarized using descriptive statistics (mean, SD). Categorical data for personal and family history were expressed as frequency and percentage of patients. Changes in clinical and laboratory parameters from baseline to the end of the study were determined using a paired-sample t-test. A predefined threshold of p<0.05 was considered statistically significant. Changes in ECG patterns across various categories were presented as the number and percentage of patients.

## Results

Baseline characteristics

A total of 250 patients were included in the study. The mean age was 46.9 years, and the average BMI was 27.7 kg/m². The average waist circumference of the included population was 94.6 cm, indicative of central adiposity, as defined by clinical thresholds. Most of the patients were male (n=147, 58.8%). The mean systolic blood pressure (SBP) was 126.9 mmHg, slightly above the normal reference range. Additionally, 31 patients (12.4%) reported alcohol consumption. A substantial proportion of patients had a family history of diabetes and hypertension. In addition to the FDC of dapagliflozin and sitagliptin, patients were prescribed concomitant antidiabetic medications; insulin was part of the regimen in one patient. Dyslipidemia (n=94, 37.6%) was the most common comorbid condition among the study population, followed by hypertension (n=80, 32.0%). Coexistence of both dyslipidemia and hypertension was reported in 35 patients (14.0%) with diabetes. Detailed demographic and baseline characteristics are presented in Table 1.

**Table 1:** Baseline characteristics of patients. Patients may have more than one comorbid condition. bpm: Beats per minute; SBP: Systolic blood pressure; DBP: Diastolic blood pressure; CVD: Cardiovascular disease; NAFLD: Nonalcoholic fatty liver disease.

Parameter	Number of patients	Percentage	Mean (SD)
Demographics and clinical characteristics			
Age (years)	250	100	46.9 (8.7)
Male	147	58.8	NA
Female	103	41.2	NA
Weight (kg)	250	100	75.5 (12.3)
Height (cm)	250	100	163.1 (9.2)
BMI (kg/m²)	250	100	27.7 (2.5)
Waist circumference (cm)	250	100	94.6 (12.1)
Respiratory rate (breaths per minute)	7	2.8	19.1 (2.1)
Pulse (bpm)	250	100	83.4 (11.0)
SBP (mmHg)	250	100	126.9 (14.3)
DBP (mmHg)	250	100	79.1 (8.0)
Personal history			
Alcohol consumption	31	12.4	NA
Family history			
Diabetes	189	75.6	NA
Hypertension	108	43.2	NA
CVD	11	4.4	NA
Comorbid conditions			
Dyslipidemia	94	37.6	NA
Hypertension	80	32	NA
Dyslipidemia and hypertension	35	14	NA
Neuropathy	20	8	NA
Hypothyroidism	16	6.4	NA
Obesity	5	2	NA
NAFLD	4	1.6	NA
Chronic kidney disease	3	1.2	NA

Treatment regimen

To reduce the risk of future complications, the FDC of dapagliflozin and sitagliptin was primarily prescribed to improve medication adherence, followed by the goal of attaining better glycemic control. This combination was prescribed to most patients (n=186, 74.7%) for more than three (3) months (Table 2).

**Table 2:** Prescription pattern of dapagliflozin and sitagliptin FDC. *: Duration of prescription data was available for 249 patients. FDC: Fixed dose combination.

Parameter	Number of patients	Percentage
Reason for prescribing FDC of dapagliflozin–sitagliptin		
Synergistic effects	13	5.2
Improve medication adherence	111	44.4
Reduce diabetic complications	37	14.8
Improve glycemic control	89	35.6
Dose strength prescribed		
Dapagliflozin 10 mg / Sitagliptin 100 mg	246	98.4
Dapagliflozin 5 mg / Sitagliptin 50 mg	2	0.8
Dapagliflozin 5 mg / Sitagliptin 100 mg	2	0.8
Duration of prescription (months)*		
1-3	63	25.3
>3	186	74.7

Changes in glycemic parameters

In the overall eligible population, statistically significant reductions were observed in HbA1c, FPG, and PPG from baseline to the end of the study (p<0.001 for all). In the subgroup of patients with hypertension or dyslipidemia, the reduction in HbA1c (1.1%) was consistent with that observed in the overall cohort and reached statistical significance. FPG and PPG levels also declined significantly in these subgroups, with the improvement in PPG being more pronounced than in FPG, indicating a greater impact on postprandial glycemic control. Table 3 presents the detailed changes in each glycemic parameter for the overall population and subgroups.

**Table 3:** Change in glycemic parameters. HbA1c: Glycated hemoglobin; FPG: Fasting plasma glucose; PPG: Postprandial plasma glucose. p-values were calculated using paired-sample t-test.

Parameter	Number of Patients	Mean (SD) Baseline	Mean (SD) End of Study	Mean Difference (95% CI)	p-value
Overall					
HbA1c (%)	250	8.4 (1.0)	7.4 (0.7)	1.1 (1.0, 1.2)	<0.001
FPG (mg/dL)	250	164.0 (49.1)	134.5 (34.1)	29.5 (24.2, 34.8)	<0.001
PPG (mg/dL)	249	226.3 (66.1)	189.5 (55.9)	36.8 (29.4, 44.2)	<0.001
Hypertension					
HbA1c (%)	80	8.4 (1.1)	7.3 (0.7)	1.1 (1.0, 1.9)*	<0.001
FPG (mg/dL)	80	168.4 (51.1)	130.4 (32.1)	38.0 (29.4, 47.0)	<0.001
PPG (mg/dL)	79	245.2 (65.7)	195.3 (61.3)	49.9 (35.5, 64.4)	<0.001
Dyslipidemia					
HbA1c (%)	94	8.5 (1.1)	7.4 (0.7)	1.1 (0.8, 1.2)	<0.001
FPG (mg/dL)	94	178.1 (57.7)	141.5 (38.0)	36.6 (27.0, 46.2)	<0.001
PPG (mg/dL)	93	242.2 (76.3)	202.1 (65.2)	40.1 (25.8, 54.7)	<0.001

Changes in renal, lipid, and clinical parameters

A statistically significant improvement of 5.3 mL/min/1.73 m² in mean eGFR (n=173) was observed from baseline to follow-up (p<0.001). Additionally, significant reductions were noted in serum creatinine (n=184) and uACR (n=121) at the end of the study visit (p<0.001 for both). The mean change in eGFR values suggested that patients transitioned from the category of 60-89 mL/min/1.73 m² to ≥90 mL/min/1.73 m² (as defined by the National Kidney Foundation). The change in TG levels among 146 patients (−23.7 mg/dL) from baseline to follow-up was statistically significant. Patients (n=194) experienced a statistically significant mean reduction of -1.7 kg in body weight (p=0.004); however, the slight reduction in BMI was not statistically significant. Reductions in SBP and diastolic blood pressure (DBP) among 215 patients at the end of the study visit were also statistically significant (p<0.001). Changes observed in these parameters are presented in Table 4. No incidences of hypoglycemia, UTIs, or genital tract infections were reported.

**Table 4:** Change in renal, lipid, and clinical parameters. eGFR: Estimated glomerular filtration rate; uACR: Urine albumin-creatinine ratio; SBP: Systolic blood pressure; DBP: Diastolic blood pressure.

Parameter	Number of Patients	Mean (SD) Baseline	Mean (SD) End of Study	Mean Difference (95% CI)	p-value
Renal Parameters					
eGFR (mL/min/1.73 m²)	173	86.7 (21.4)	92.0 (19.7)	-5.3 (-6.8, -3.7)	<0.001
Serum creatinine (mg/dL)	184	1.4 (0.7)	0.9 (0.3)	0.5 (0.4, 0.6)	<0.001
uACR (mg/g)	121	173.3 (283.0)	96.9 (131.1)	76.5 (45.5, 107.4)	<0.001
Lipid Parameters					
Serum triglycerides (mg/dL)	146	190.5 (123.7)	166.8 (85.9)	23.7 (9.1, 38.3)	0.002
Clinical Parameters					
Weight (kg)	194	76.2 (13.4)	74.5 (12.7)	-1.7 (-2.3, -1.1)	<0.001
BMI (kg/m²)	196	27.9 (3.3)	27.8 (2.7)	0.1 (-0.2, 0.4)	0.433
SBP (mmHg)	215	130.5 (16.1)	124.5 (13.4)	5.9 (4.2, 7.7)	<0.001
DBP (mmHg)	215	80.2 (7.7)	77.8 (8.1)	2.3 (1.9, 3.5)	<0.001

## Discussion

This real-world evaluation assessed the impact of early initiation of the FDC of dapagliflozin and sitagliptin on glycemic profile, renal function, lipid profile, and BP in Indian patients with T2DM. Statistically significant improvements were observed in key clinical parameters, including glycemic control, renal function, lipid levels, BP, and body weight. These results support the role of early initiation of this FDC in attaining better metabolic control and mitigating long-term cardiovascular and renal complications.

The demographic profile of the study population was closely aligned with previously reported Indian studies. The mean age of patients (n=250) in this study was 46.9±8.7 years, which is comparable to the mean age of 51.14±5.55 years reported in a study including T2DM patients aged 18 to 59 years [15]. Male predominance (n=147, 58.8%) observed in the cohort also echoed the gender distribution presented by Chawla M et al. (77.74% males and 22.26% females) [15]. Notably, the mean BMI of 27.7 kg/m² and the mean waist circumference of 94.6 cm indicated generalized and central adiposity, which are key contributors to insulin resistance, especially in the Indian context [16].

Family history remains a strong risk factor for developing diabetes, and more than three-quarters of the study population reported such a history [17]. Dyslipidemia and hypertension were the most common conditions coexisting with T2DM, and 35 patients (14.0%) had a triad of diabetes, dyslipidemia, and hypertension. These patients belong to the high-risk category, predisposed to future renal and cardiovascular complications. This underscores the need for the selection of antidiabetic agents that act beyond glycemic control [18].

Adherence to medications is a key factor for achieving optimal glycemic control. A meta-analysis by Wei Q et al. reported a 1.29-fold improvement in compliance with FDCs compared to free-equivalent combinations (FEC) [19]. Similarly, Benford et al. reported that the prescription of DPP-4-based FDCs was positively associated with compliance and glycemic control. In line with this, the primary reason for the prescription of the FDC of dapagliflozin and sitagliptin in our cohort was to improve adherence, followed by achieving better glycemic control.

The magnitude of HbA1c reduction observed in our study (1.1%) is comparable to the 1.05±0.83% decrease reported by Chawla M et al. over a 12-week period. Improvements in FPG and PPG also align well with the published literature [15], reflecting the glycemic effectiveness of this FDC in clinical practice. Reductions in HbA1c were consistent (1.1%) in patients with coexisting conditions of dyslipidemia or hypertension. Sitagliptin, through its incretin-mediated mechanism, is known to have a greater effect on postprandial blood glucose (PPBG) compared to fasting blood glucose (FBG). In a two-year study, the addition of sitagliptin to existing antidiabetic therapy reduced FBG by 12.7% and PPBG by 20.5% [20]. Given that elevated PPBG has been independently associated with cardiovascular risk, targeting PPBG may offer additional benefits in preventing vascular complications in patients with T2DM [21].

Favorable changes were observed in the renal parameters assessed in the present study, including eGFR, serum creatinine, and uACR. These findings are aligned with the BRIDGE-DS study conducted by Maiti A et al., which reported the renoprotective effects of the FDC of dapagliflozin and sitagliptin, including reduced serum creatinine and improved eGFR [22]. Additionally, DPP-4 inhibitors exert antiproteinuric effects, and SGLT-2 inhibitors, when used over a prolonged period, help prevent the progression of chronic kidney disease (CKD) [23]. The observed reduction in triglyceride levels further supports the cardiovascular protective potential of this FDC [24].

The modest but statistically significant weight reduction observed in our study is consistent with existing evidence. Dapagliflozin promotes moderate weight loss in overweight and obese patients through its glycosuria mechanism, while sitagliptin enhances incretin hormone activity, contributing to modest weight reduction. The reduction observed in the present study exceeded that reported by Kovil R et al. [25]. This improvement in weight is expected to contribute to better cardiometabolic outcomes. Furthermore, the Real DAPSI study demonstrated the multifaceted benefits of the FDC of dapagliflozin and sitagliptin, highlighting its role in cardiovascular and renal protection, weight reduction, and blood pressure management. Although the extent of BP reduction reported in the current study was lower than that in the Real DAPSI study, the reduction achieved statistical significance, underscoring its clinical relevance [26].

Importantly, no incidences of hypoglycemia or urinary/genital tract infections were reported. These findings are consistent with the safety data from Lukka PB et al., who demonstrated that this FDC is well tolerated [27]. The low incidence of genitourinary events may be attributed to the interaction of DPP-4 and SGLT-2 proteins at the renal tubular-cell membrane level, or the inhibition of the DPP-4 enzyme in certain pathogenic microorganisms, rendering them inactive [28]. The glucose-dependent mechanisms of both agents also contribute to the minimal risk of hypoglycemia. Dapagliflozin facilitates urinary glucose excretion when plasma glucose levels are elevated, while sitagliptin stimulates insulin secretion and suppresses glucagon release [29]. Thus, the FDC of dapagliflozin and sitagliptin is endorsed as a well-tolerated option for treatment intensification, especially in patients with multiple comorbid conditions [23].

The present study reflects actual clinical practice, including a diverse patient population with multiple comorbidities from different demographic and geographic backgrounds. This provides insights into the clinical effectiveness of the combination beyond the controlled settings of randomized clinical trials.

However, the retrospective design of the study carries the inherent limitation of missing data. The study did not include a comparative group, and being a real-world study, there was no control over potential confounding factors. Additionally, the relatively short follow-up period restricts the evaluation of long-term effectiveness and safety. These factors should be considered when interpreting the results.

## Conclusions

This real-world, retrospective evaluation demonstrated the clinical utility of early initiation of the FDC of dapagliflozin and sitagliptin in Indian patients with T2DM and associated cardiometabolic comorbidities. The combination led to significant improvements in glycemic control (HbA1c, FPG, and PPG), renal parameters (eGFR, serum creatinine, and uACR), lipid profile (serum triglycerides), body weight, and blood pressure (systolic and diastolic), with a favorable tolerability profile (no adverse events). The high prevalence of central obesity and metabolic comorbidities in the study population underscores the need for therapeutic agents that provide comprehensive metabolic benefits. The present findings support the role of this FDC in holistic metabolic management in routine clinical practice. Further prospective studies with longer follow-up are warranted to validate these outcomes and assess long-term effectiveness and safety.

## Figures and Tables

**Figure 1:**
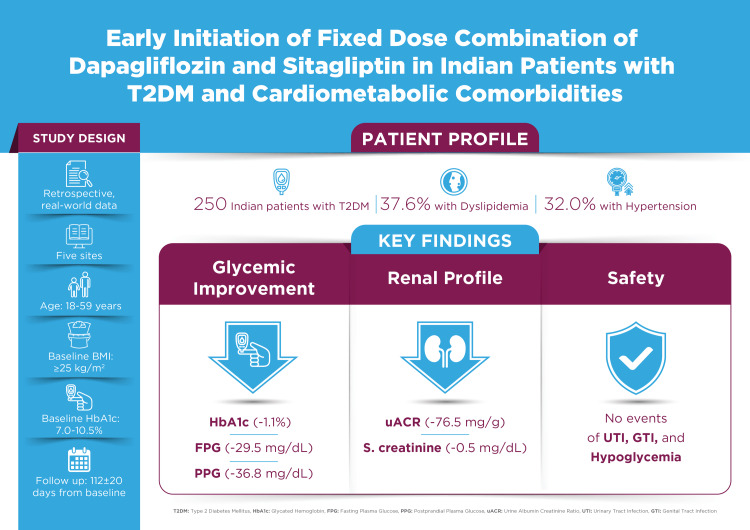
Study at a glance. T2DM: Type 2 diabetes mellitus; HbA1c: Glycated hemoglobin; FPG: Fasting plasma glucose; PPG: Postprandial plasma glucose; uACR: Urine albumin-creatinine ratio; GTI: Genital tract infection.

## References

[1] Maniyara K, Kodali PB (2025). Assessing type-2 diabetes risk based on the Indian diabetes risk score among adults aged 45 and above in India. Sci Rep.

[2] Kalra S, Singh A, Das S (2025). Sitagliptin as an add-on therapy to other glucose-lowering agents in patients with type 2 diabetes mellitus: a narrative review. J Assoc Physicians India.

[3] Gupta RD, Kothadia RJ, Parray AA (2023). Association between abdominal obesity and diabetes in India: findings from a nationally representative study. Diabet Epidemiol Manag.

[4] Jayagopal PB, Rao MS, Vijaykumar R (2019). Consensus statement for the management of dyslipidemia and hypertension in the Indian population with diabetes. Int J Adv Med.

[5] Davies MJ, Aroda VR, Collins BS (2022). Management of hyperglycemia in type 2 diabetes, 2022. A consensus report by the American Diabetes Association (ADA) and the European Association for the Study of Diabetes (EASD). Diabetes Care.

[6] American Diabetes Association Professional Practice Committee (2024). 9. Pharmacologic Approaches to Glycemic Treatment: Standards of Care in Diabetes. Diabetes Care.

[7] Kanumilli N, Brunton S, Cos X, Deed G, Kushner P, Lin P, Nolte J (2021). Global survey investigating causes of treatment inertia in type 2 diabetes cardiorenal risk management. J Diabetes Complications.

[8] Sivadas A, Sahana S, Jolly B (2024). Landscape of pharmacogenetic variants associated with non-insulin antidiabetic drugs in the Indian population. BMJ Open Diabetes Res Care.

[9] Gaggini M, Sabatino L, Suman AF, Chatzianagnostou K, Vassalle C (2025). Insights into the roles of GLP-1, DPP-4, and SGLT2 at the crossroads of cardiovascular, renal, and metabolic pathophysiology. Cells.

[10] Sakamoto Y, Oyama J, Ikeda H (2013). Effects of sitagliptin beyond glycemic control: focus on quality of life. Cardiovasc Diabetol.

[11] Gan S, Dawed AY, Donnelly LA, Nair AT, Palmer CN, Mohan V, Pearson ER (2020). Efficacy of modern diabetes treatments DPP-4i, SGLT-2i, and GLP-1RA in White and Asian patients with diabetes: a systematic review and meta-analysis of randomized controlled trials. Diabetes Care.

[12] Cho YK, Kang YM, Lee SE (2018). Efficacy and safety of combination therapy with SGLT2 and DPP4 inhibitors in the treatment of type 2 diabetes: a systematic review and meta-analysis. Diabetes Metab.

[13] Arya DS, Chowdhury S, Chawla R (2019). Clinical benefits of fixed dose combinations translated to improved patient compliance. J Assoc Physicians India.

[14] Ayyar V, Ommen T, Shukla RP (2025). Synergizing sodium-dependent glucose transporter inhibitors with dipeptidyl-peptidase 4 inhibitor and metformin: a novel approach to diabetes management in India. Int J Basic Clin Pharmacol.

[15] Chawla M, Panneerselvam D, Gundgurthy A (2024). Retrospective observational study on assessing sitagliptin and dapagliflozin as a fixed-dose combination in the Indian population with type 2 diabetes mellitus: the SIDAXA study. Cureus.

[16] Shah A, Kanaya AM (2014). Diabetes and associated complications in the South Asian population. Curr Cardiol Rep.

[17] Scott RA, Langenberg C, Sharp SJ (2013). The link between family history and risk of type 2 diabetes is not explained by anthropometric, lifestyle or genetic risk factors: the EPIC-InterAct study. Diabetologia.

[18] Chen SC, Tseng CH (2013). Dyslipidemia, kidney disease, and cardiovascular disease in diabetic patients. Rev Diabet Stud.

[19] Wei Q, Zhou J, Li H, Wang L, Wu Y, Ma A, Guan X (2023). Medication adherence with fixed-dose versus free-equivalent combination therapies: systematic review and meta-analysis. Front Pharmacol.

[20] Derosa G, Ragonesi PD, Fogari E (2014). Sitagliptin added to previously taken antidiabetic agents on insulin resistance and lipid profile: a 2-year study evaluation. Fundam Clin Pharmacol.

[21] Node K, Inoue T (2009). Postprandial hyperglycemia as an etiological factor in vascular failure. Cardiovasc Diabetol.

[22] Maiti A, Pandey AK, Kale S (2025). The BRIDGE-DS study: improved glycemic control and renal function in type 2 diabetes mellitus patients using the fixed-dose combination of dapagliflozin and sitagliptin. J Endocrinol Metab.

[23] Ray S, Ezhilan J, Karnik R (2024). Expert opinion on fixed dose combination of dapagliflozin plus sitagliptin for unmet cardiovascular benefits in type 2 diabetes mellitus. JODB.

[24] Aberra T, Peterson ED, Pagidipati NJ (2020). The association between triglycerides and incident cardiovascular disease: What is "optimal"?. J Clin Lipidol.

[25] Kovil R, Deshmane R, Bharathi BP (2024). Real-world effectiveness of dapagliflozin and sitagliptin fixed-dose combination in indian patients with type 2 diabetes: a retrospective analysis of electronic medical records stratified by BMI. EJEA.

[26] Bhattacharjee R, Rai M, Joshi P, Prasad A, Birla A (2023). The real DAPSI: a real-world retrospective study on assessing the efficacy and safety of a fixed-dose combination of dapagliflozin and sitagliptin in the Indian population. Cureus.

[27] Lukka PB, Tang W, Hammarstedt A, Conrad T, Heijer M, Karlsson C, Boulton DW (2024). Racial comparison of the pharmacokinetics and safety of fixed-dose combination of dapagliflozin/sitagliptin in Western and Korean healthy adults. Clin Ther.

[28] Chadha M, Das AK, Deb P (2022). Expert opinion: optimum clinical approach to combination-use of SGLT2i + DPP4i in the Indian diabetes setting. Diabetes Ther.

[29] Kalra S, Kesavadev J, Chadha M, Kumar GV (2018). Sodium-glucose cotransporter-2 inhibitors in combination with other glucose-lowering agents for the treatment of type 2 diabetes mellitus. Indian J Endocrinol Metab.

